# Gear-like rotatable optical trapping with radial carpet beams

**DOI:** 10.1038/s41598-020-68695-8

**Published:** 2020-07-16

**Authors:** Jamal Bayat, Faegheh Hajizadeh, Ali Mohammad Khazaei, Saifollah Rasouli

**Affiliations:** 1 Department of Physics, Institute for Advanced Studies in Basic Sciences (IASBS), Zanjan, 45137-66731 Iran; 2 Optics Research Center, Institute for Advanced Studies in Basic Sciences (IASBS), Zanjan, 45137-66731 Iran

**Keywords:** Optics and photonics, Physics

## Abstract

Optical tweezers have become a powerful tool in the fields of biology, soft condensed matter physics, and nanotechnology. Here, we report the use of recently introduced radial carpet beams (RCBs) in the optical tweezers setup to trap multiple particles. An RCB is produced by diffraction of a plane or Gaussian beam from an amplitude radial grating. Because of the radial symmetry of the grating, all the diffraction orders are propagated along the optical axis and are used for trapping. Based on the number of grating spokes, the produced RCB has a definite number of high-intensity spots on the transverse plane located over a circular ring. These high-intensity spots of the beam provide multi-traps when it passes through an objective lens and have enough gradient force to trap polystyrene and silica particles. Moreover, the diffracted light from the grating has this property to transfer the angular momentum. We show that the multi-trapped birefringent particles could rotate in their own traps when polarization of the trapping RCB to be circular. In addition, the orbital rotation of the particles is simply executable by manually rotating the grating in its plane around the optical axis.

## Introduction

One half of the Nobel Prize in Physics 2018 has been awarded to Arthur Ashkin for his invention of ’optical tweezers’ that is widely used in manipulating micro- to nanoparticles, atoms, molecules, and biological cells. Arthur Ashkin reported the detection of optical scattering and gradient forces on micron sized particles in 1970^1^. A major success took place in 1987 when Ashkin used tweezers to capture living bacteria without sacrificing them^2,3^. In the optical tweezers, the optical pressure of the light beam provides the force that is necessary for the particle movement^4^. So far, many scientific insights were obtained from the manipulation of microscopic systems using single or two discrete beams optical traps^5,6,7,8,9,10,11,12,13^. However, converting the single beam optical tweezers into multifunctional optical traps by grabbing a complex system at different points simultaneously, would provide valuable applications such as: nanofabrications, investigation of interacting colloidal particles in a potential energy pattern, and optical actuators for lab-on-a-chip technologies^14^. Multiple optical traps can be created by various approaches such as optical holography^15,16,17^, by a single fast scanning trap integrated with the bilateral confocal scanning laser microscope^18^, or using a phase mask which needs a careful adjustment of the optical setup^19^.

A general alternative to generate multiple-beam optical tweezers is to use Spatial Light Modulators (SLM) such as Digital Micro Devices (DMD) and Liquid crystal SLM to modify the laser wavefrom^20^. They significantly expand the potentials of multiple trapping by a single beam even for single neutral atoms^21^, however generating large numbers of the traps typically result in low diffraction efficiency and unwanted diffraction orders^22^.

The usage of light diffraction for beam shaping could be considered in another way. For instance, the diffraction of plane and Gaussian beams from radial gratings presents interesting physical effects. As recently proposed, the diffraction of a plane wave from radial amplitude and/or phase gratings reveals a complete set of radial beams so called “Combined half-integer Bessel-like beams” where in some special cases they named “radial carpet beams (RCB)”^23^. In a couple of recently published works the theory of diffraction of a plane wave from radial gratings has been presented both by the Fresnel integral and wave equation methods. We also formulated the diffraction of vortex beams from radial gratings and proposed an intensity-based method for wavelength alteration and optical communication^24^. The RCBs were used for information transmission^25^. In another work by adding an azimuthally periodic term, say a radial phase grating function, into the argument of a linear phase grating, generation of varied RCBs over different diffraction orders of the linear phase grating with controlled intensity sharing among the generated RCBs was investigated^26^.

In this Letter, we introduce a simple, high-efficient, low-cost, and reliable method for annular multiple trapping in 2D via diffraction of a Gaussian beam from an amplitude radial grating. Simply, by rotating the radial grating, the resulted multiple traps rotate around the optical axis, simultaneously.

## Methods

Here we briefly present the theoretical approach on the diffraction of a Gaussian beams from radial gratings and the experimental details of the optical setup.

### Theoretical approach

**Figure 1:**
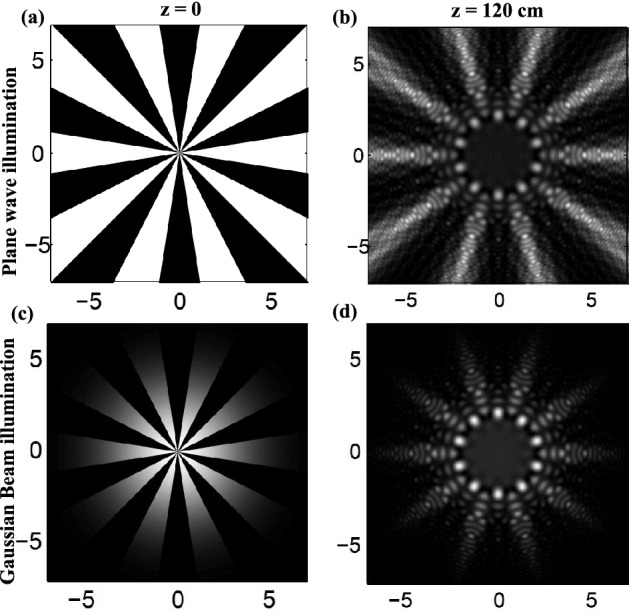
(**a**) The intensity pattern immediately after an amplitude radial grating having binary profile with 10 spokes illuminated by a plane wave and (**b**) the calculated intensity of the resulted RCB at propagation distance 120 cm. (**c**, **d**) The same patterns when a Gaussian beam having a radius of 2 mm illuminates the same radial grating. All lengths are in millimeters.

The theory of diffraction of plane and Gaussian beams from radial gratings are presented in Refs.^23,24^. For an amplitude radial grating with binary profile having $m = 10$ spokes illuminated by a plane wave or Gaussian beam the calculated intensity pattern immediately after the grating and the corresponding resulted radial carpet patterns at a propagation distance 120 cm are shown in Fig. 1. On the diffraction patterns there are *m* main intensity spots around a circle surrounding the patternless central area. As is apparent, for the case of Gaussian beam, the intensity of the main lobes are dominate and at the same time the intensity of the lateral spokes drops to zero as the radius increases. This feature candidates the use of such diffraction pattern for multiple trapping.

### Experimental setup

**Figure 2:**
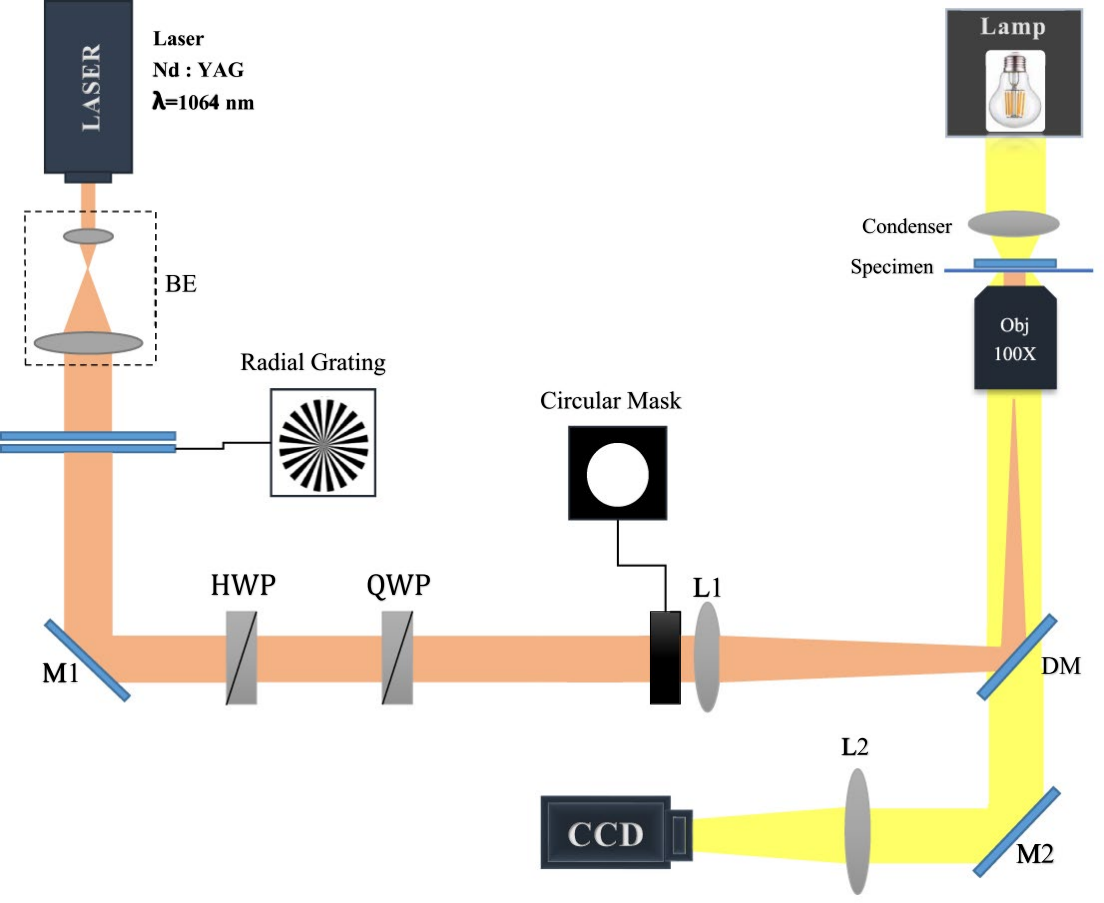
Experimental setup; BE, M, HWP, QWP, L, DM, and QPD are the beam expander, mirror, half and quarter wave plates, lens, dichroic mirror, and quadrant photo diode, respectively.

The used optical tweezers setup, schematically shown in Fig. 2, is implemented on an inverted microscope (Olympus IX71). The trapping laser beam is a near-infrared laser ($\lambda = 1064\hbox { nm}$, Coherent). The laser beam, first is expanded and collimated by a telescopic system, and impinges on an amplitude radial grating having binary profile and 10, 15, 20, or 50 spokes. The amplitude radial gratings are simply printed on plastic papers. The beam waist on the grating was about 6 mm. At a distance 140 cm from the grating, resulted petal-like light field passes through an annular aperture, and a very clear petal beam is produced. It should be noted that, unlike the case of plane wave illumination of the radial gratings, in the diffraction of a Gaussian beam from a radial grating the resulted diffraction pattern changes very slowly under propagation. This effect does not appear in small paths such as the length of the laser path before the objective. With the aid of a positive lens with 50 cm focal length and a high-NA objective (Olympus, 100 ×, oil immersion, NA = 1.3) in confocal arrangement, the diameter of the resulted petal beam reduces. This positive lens focuses the laser beam onto the back pupil of the microscope objective, therefore the laser beam is collimated inside the sample chamber. The diffracted pattern coupled into the optical path of the microscope using a dichroic mirror. To maintain polarization of the beam in circular state after passing through dichroic mirror, we used a set of half and quarter wave plates to compensate the polarization change occurred by the dichroic mirror on the beam. The collimated petal beam with reduced diameter passes through the chamber. The sample chamber was implemented at the focal plane of the objective. The chamber was mounted on a piezo stage (PI-527.2cl, Physik Instrumente) which allows nanoscale lateral positioning. The sample chamber was made by a coverslip and a microscope slide, spaced by means of two stripes of a double-sided tape. The particles used in this work were polystyrene beads (Bangs Lab.) with mean diameters of 1.09 μm, 2.54 μm silica particles, and up to 6 μm vaterite particles. Successive images of the trapped particles were recorded by a CCD camera and stored in a computer.

## Result

### The use of radial carpet beams in multiple trapping

**Figure 3:**
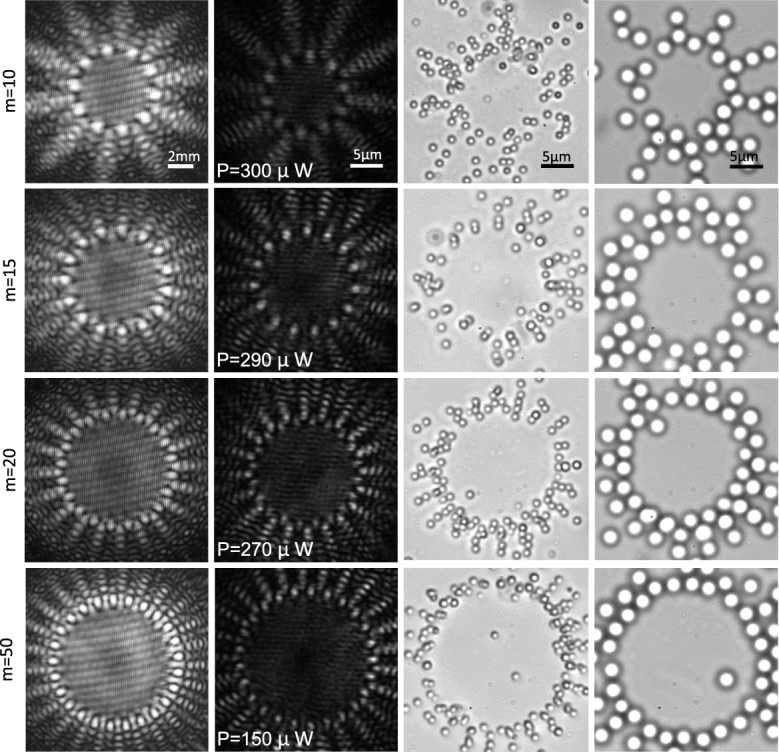
Generated radial carpet patterns by the diffraction of a Gaussian beam from different radial amplitude gratings having binary profile and different spoke numbers at a distance 120 cm from the grating (first column) and at the focus of the objective lens (second column). Different images of the trapped polystyrene particles with a diameter of 1.09 μm (third column) and 2.54 μm silica particles (forth column). The laser power in each individual trapping spot at the sample is indicated in the second column.

In Fig. 3 the experimentally produced radial carpet patterns, say the multi-traps, at the input plane of L1 lens in Fig. 2 (first column) and at the focal plane of the objective lens (second column) are illustrated. The third column shows the trapped polystyrene particles in two dimensions with a diameter of 1.09 μm in the vicinity of the top surface of the sample chamber (because the particles are suspended in the aqueous medium, and as the laser beam pushed them upwards, the particles were attached to the upper wall of the chamber). The fourth column shows 2D trapping images of 2.54 μm silica particles at the chamber floor. Silica particles settled at the bottom of the chamber due to their high density. It should be noted that, by increasing the laser power, the scattering force can overcome to the weight force and particles can be pushed to the upper wall. Here, high concentration samples of the particles were prepared due to the need for a number of particles to be trapped.

**Figure 4:**
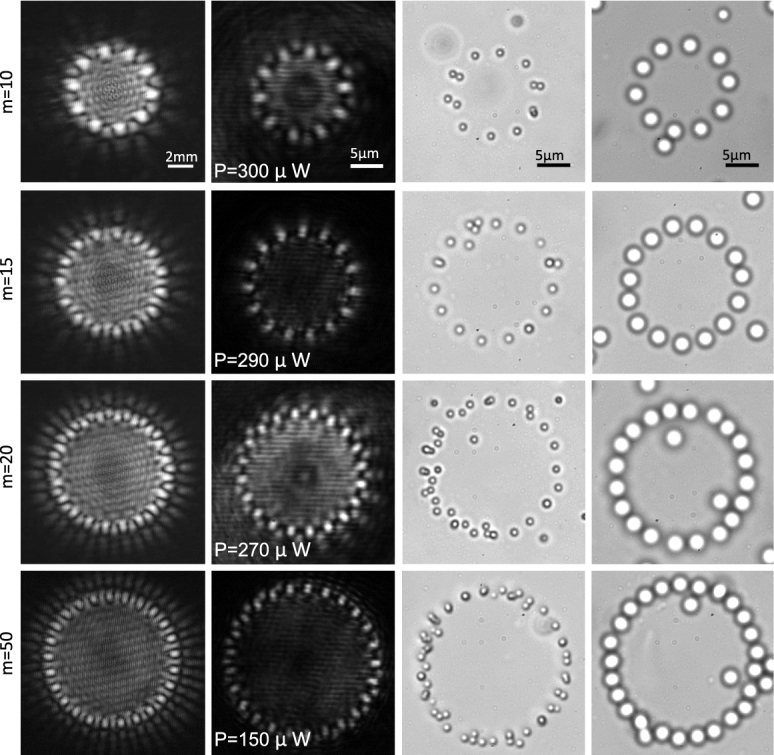
The same patterns and trap images of Fig. 3 when a stop circular aperture was used before the L1 lens shown in Fig. 2. The laser power in each individual trapping spot at the sample is indicated in the second column. See also Visualizations 1 and 2 that they show tapping and annular rotating of the trapped particles.

By inserting a circular aperture after L1 lens, shown in Fig. 2, we allow only the main spots of the radial carpet pattern enter to the trapping system. This guarantees to have a very clear ring pattern inside the chamber. In Fig. 4, the same patterns and trap images of Fig. 3 are shown when a stop circular aperture was used before L1 lens. In this case, it is seen that only the main spots of the generated radial carpet patterns pass through the aperture and the particles trapped on the annular ring. By implementing the grating on a rotatable holder, we rotate the grating around its axis, and as a result the diffracted carpet pattern and trapped particles rotate around the beam axis, manually. Visualizations 1 and 2 show tapping and annular rotating of 2.54 μm silica particles in 15 intensity main spots. The video speed is three times faster than the real-time. As could be observed in the videos, each particle was trapped in one laser spot and the rotation speed of the grating is such that the particles follow their own trap and do not jump between neighboring potential wells. The threshold of particle escape from a trap occurs at a frequency of about 0.12 Hz. The laser power of the beam in visualizations 1 and 2 is about 1.6 *mW* per each spot.

**Figure 5:**
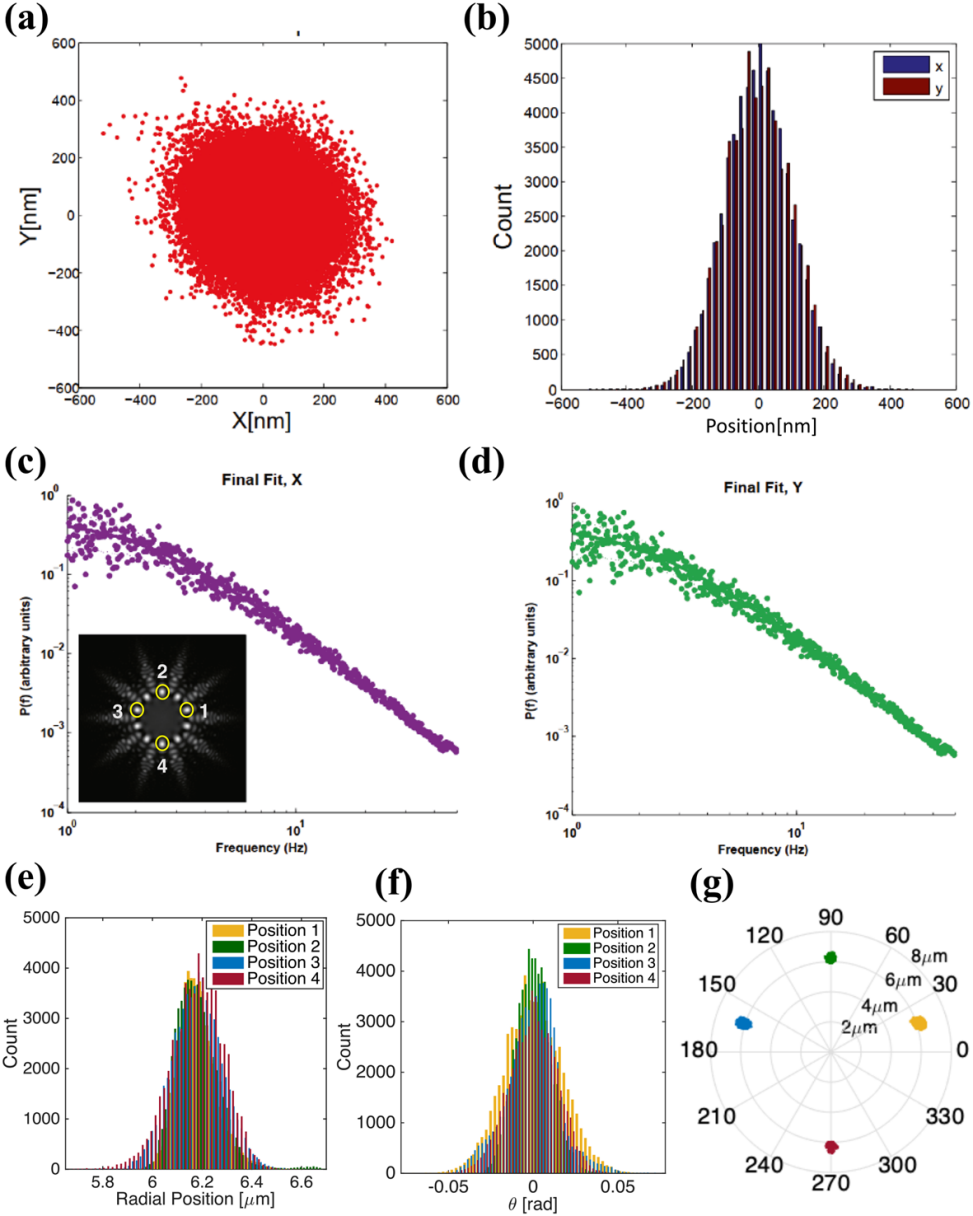
(**a**) CCD-measured center of the mass trajectory and (**b**) position distribution of a trapped 2.54 μm silica bead during a time interval of 10 mins. (**c**, **d**) show the corresponding power spectra in *x* and *y* directions, respectively. The inset shows locations of four selected traps in the radial carpet pattern with 10 main spots, used for calibrating the trapping stiffness. Distribution of radial position (**e**) and tangential position (**f**) of the trapped particles in positions 1–4. (**g**) Position distribution of the similar trapped particles in polar display.

Successive images of the trapped particles in a time interval 10 minutes were recorded and used to estimate the stiffness of the optical traps. The frame rate of the CCD camera was 100 fps and 60,000 images were taken from each trap. Using MATLAB software, the center of mass of four trapped particles shown in the inset of Fig. 5c were determined at successive frames. We estimate the stiffness of the traps by determining the corner frequencies of the power spectra of the particles positions. Figure 5a shows the distribution of the center of mass of the particle 1, shown in the inset of Fig. 5c, in Cartesian coordinates. The size of the trapped silica particles was 2.54 microns. In Fig. 5b, the histogram of the center of mass of particle 1 in two directions are shown. Figure 5c and d show the corresponding power spectra in two perpendicular directions. In the last row, Fig. 5e, represents the radial distribution of the trapped particle relative to the center of the ring for positions 1–4. Figure 5f shows the tangential distribution of the center of mass for the trapped particles at the positions 1–4 in relative to their equilibrium locations. Figure 5g represents the distribution of the similar trapped particles in polar coordinates.

We estimate the trap stiffness using power spectrum of the displacement distributions for the particles trapped in four distinct intensity spots marked with yellow circles in the inset of Fig. 5c. Each experiment was repeated two times and the mean value of trap stiffness 0.35 $\frac{fN}{nm}$ is obtained using a MATLAB code^27^. The total power of the radial beam at the sample (after objective) was 37 mW and the share of each spot was about 3.7 mW. In addition to the power spectrum analysis, the width of the position distribution is used to calibrate the optical trap by the equipartition theorem. The trapping stiffness obtained by the equipartition theorem has less than 21% different, compared to the power spectrum method.

The angular momentum transferred by circularly polarized beam is able to rotate an optically trapped birefringent particle^28^. Here we investigate multiple trapping of the birefringent particles, vaterite particles, and observed the rotation of the trapped particles in their own locations when we changed polarization of the produced radial carpet pattern to a circular polarization. For this purpose, we used a pair of quarter and half wave plates after the radial grating in Fig. 2. Polarization of the laser beam was almost linear and after passing through the grating, as it has inhomogeneous structure, the beam gets a bit ellipticity. Visualization 3 shows axially rotation of trapped vaterite particles in their own locations by circularly polarized radial carpet beam. The power of each of the main spots containing the tail is about 4.5 milliwatts inside the chamber. In this visualization, the trapped vaterite particles, rotate at different frequencies due to their different geometry and size. For instance, there is a relatively large vaterite particle in the left side of the ring with an approximate diameter of 6 μm which rotates at a frequency of about 0.4 Hz, like a pyramidal particle which is trapped in the 30th second with the size of about 3 μm in the right. In contrast, under the same conditions a cube-shaped particle trapped at 56th second in the bottom of the ring has a rotation frequency of about 2 times faster (0.8 Hz). Movie speed in the visualization 3 is two times faster than in real-time.

## Discussion

Below, we describe what we believe is the first experimental demonstration of the RCB applied to the trapping of multiple particles in an optical tweezer system, and compare our method with the other diffraction-based methods that generally use pixelated SLM devices to generate a desired phase or amplitude aperture. In principal, the far-field diffraction pattern of an aperture includes several intensity spots that used for multiple trapping. As an SLM has a periodic structure in two directions, when a given phase or amplitude structure is imposed on it and a collimated laser beam illuminates that, the far-field diffraction pattern (or equally the spectrum) of the imposed aperture is replicated by the impulses of the SLM^26^. Each impulse corresponds to a diffraction order of the SLM. In other words, rather than producing a copy of the desired diffraction pattern, it is replicated over all diffraction orders of the SLM. Indeed, when generating large arrays of traps with SLMs, this feature reduces the diffraction efficiency. Moreover, generating unwanted zero-order and higher-orders accompanying any desired diffraction pattern are clearly issues, while one diffraction order of the SLM is usually utilized for multiple trapping^22^. In contrast, in the diffraction of a plane or Gaussian wave from an amplitude radial grating, there is no different diffraction orders and the entire diffracted beam is propagated around the optical axis. The diffraction pattern has a shape-invariant form under propagation, its divergence is less than the divergence of the conventional beams like a Gaussian beam, and its divergence is also less than the divergence of the far-field diffraction from conventional apertures. Due to these features, the resulted diffraction pattern has been called ’nondiffracting and accelerating RCB’. These features provide the use of entire transmitted power of the laser beam through the radial grating (50%) for multiple trapping. (For further details, see Refs.^23,24^). Moreover, the SLM-based multiple traps not only suffer from the pixel diffraction problems and require a specific optical alignments, they are also partly expensive. In contrast, the proposed method for creating multiple traps is low cost and the needed radial grating could be simply designed with the most computer softwares and printed on a piece of glass or a plastic paper with a commercial printer. Optically trapping and rotating particles in a gear-like/ring patterns could have a significant impact on a variety of contexts such as producing micromechanics^29^ and assembling a micro-gear/ring with the desired number of particles to produce mechanical micro-optic pumps^30^. Optical trapping and rotation on a ring can force even large particles to spin and can be used for manipulation living cells^31^.

## Conclusion

In summary, we have successfully used the radial carpet patterns produced in the diffraction of a Gaussian beam from a binary amplitude radial grating for multiple trapping of particles over an annular array, in which all the trapped particles can rotate together over the optical axis by rotating the radial grating over its axis, manually. We also show that if polarization of the produced radial carpet light field to be circular, the trapped birefringent particles will rotate in their own traps. This method of multiple trapping is very simple, low cost, and does not need complicated devices such as spatial light modulators (SLM)^32,33,34,35^ or fast scanning devices^18^. In comparing with the SLMs, as the diffraction pattern from a radial grating is not divided into different diffraction orders, here working with low power lasers is possible. We believe that our findings would have significant impact on the multiple optical trapping. Another candidate for multiple trapping and manipulating of the particles in a 2D array of intensity spots is the use of diffraction of a vortex beam from 2D gratings in the Cartesian coordinates^36^. This work is under study and results will appear elsewhere.

## Supplementary information

Supplementary Video 1.

Supplementary Video 2.

Supplementary Video 3.
